# Exploratory Analysis of Nationwide Japanese Patient Safety Reports on Suicide and Suicide Attempts Among Inpatients With Cancer Using Large Language Models

**DOI:** 10.1002/pon.70150

**Published:** 2025-05-04

**Authors:** Ken Kurisu, Maiko Fujimori, Saki Harashima, Masako Okamura, Kazuhiro Yoshiuchi, Yosuke Uchitomi

**Affiliations:** ^1^ Department of Cancer Survivorship and Digital Medicine Jikei University School of Medicine Tokyo Japan; ^2^ Division of Survivorship Research National Cancer Center Institute for Cancer Control Tokyo Japan; ^3^ Department of Stress Sciences and Psychosomatic Medicine Graduate School of Medicine The University of Tokyo Tokyo Japan

**Keywords:** BERTopic, cancer, GPT‐4o, large language models, oncology, open AI, patient safety, psycho‐oncology, suicide

## Abstract

**Objective:**

Patients with cancer have a high risk of suicide. However, evidence‐based preventive measures remain unclear. This study aimed to investigate suicide prevention strategies for hospitalized patients with cancer by analyzing nationwide patient safety reports using large language models.

**Methods:**

Data were drawn from patient safety reports collected by the Japan Council for Quality Health Care from 620 hospitals. Reports involving suicides or attempts among patients with cancer were analyzed. BERTopic was used to identify topics in free‐text reports, and conditions such as depressive symptoms were labeled using the OpenAI API. Logistic regression was conducted to analyze the relationship between pre‐incident conditions and proposed countermeasures.

**Results:**

Among 213 reports, key topics included mental and physical distress, symptom deterioration, nursing records, and post‐incident documentation. Over 40% of patients exhibited depressive symptoms, and 30% expressed suicidal ideation. However, fewer received specialized mental care. Notably, over 10% appeared to experience delirium, potentially contributing to the incident. The most frequently suggested countermeasures were mental distress treatment, enhanced medical staff communication, and improved information sharing with families. Logistic regression revealed several associations between proposed countermeasures and pre‐incident conditions, including mental health intervention for patients without prior treatment, physical interventions for those in severe pain, and improved staff communication for those with depressive symptoms.

**Conclusions:**

This study, based on nationwide patient safety reports, highlights critical suicide prevention strategies for hospitalized patients with cancer, many of which align with previously proposed strategies. Additionally, the study provides new insights, such as the need for preventive measures to manage delirium.

## 1 Background

Numerous studies have shown that patients with cancer are at a significantly higher risk of suicide compared to the general population [1]. This increased risk has also been documented in Japan through the National Cancer Registry, the country's only comprehensive national cancer database [2]. Furthermore, regional variations in suicide risk have been observed [3]. Data from the Tokyo Medical Examiner's Office, which covers more than 10% of Japan's population, indicate that 4.2% of suicides occur within hospitals [4, 5], where many cases could be preventable.

Designated Cancer Care Hospitals in Japan are required to implement suicide prevention strategies [6], and guidelines have been developed for this purpose [5]. However, effective interventions to prevent suicide among patients with cancer remain unclear [7]. Although inpatient suicides are relatively rare, they cause substantial psychological distress for both medical staff and the patient's families [8], highlighting the need for effective preventive measures.

A systematic review identified cancer as the predominant physical disease associated with suicides in medical settings [9]. A previous study in Japan found that patients with cancer accounted for approximately half of all suicides in medical settings, based on hospital questionnaires [10]. The study also revealed the prevalence of suicidal ideation and psychiatric comorbidities before the incidents [10]. Another study, which analyzed patient safety reports of suicide among patients with cancer, identified root causes such as inadequate psychosocial assessment and poor communication among healthcare teams [11]. However, these reports provided limited information regarding pre‐incident conditions and preventive countermeasures.

The Japan Council for Quality Health Care publicly shares patient safety reports submitted by hospitals across Japan on its website [12]. These reports contain details of incidents and suggestions from medical staff on potential countermeasures in the form of unstructured free‐text narratives, which complicate data analysis. However, the recent advancement of large language models, such as ChatGPT, has enabled efficient and accurate processing of unstructured texts. These models have demonstrated accuracy comparable to human performance in extracting medical information from radiology reports and pharmaceutical documents, as well as in diagnosing mental disorders [13, 14, 15, 16, 17, 18, 19, 20].

This study aimed to investigate the circumstances surrounding suicides among hospitalized patients with cancer, as well as those with other physical illnesses, and to identify effective preventive countermeasures by analyzing nationwide Japanese patient safety reports using large language models.

## 2 Methods

### 2.1 Ethics Considerations

This study used anonymized data that is freely available online. Therefore, the research ethics guidelines are not applicable to this study, and informed consent was not required.

### 2.2 Data Source

This study used medical incident reports made publicly available on the website of the Japan Council for Quality Health Care [12]. These reports included: (1) mandatory submissions as per the Regulations for Enforcement of the Medical Practitioners Act from university hospitals, the National Research and Development Agency, the National Hospital Organization, and advanced treatment hospitals across Japan (122 institutions as of December 31, 2023), and (2) voluntary submissions from other medical institutions (498 institutions as of December 31, 2023).

We extracted reports submitted between 2010 and 2023 that involved suicides or suicide attempts by patients with cancer. Reports categorized under “patient suicide” or “suicide attempt” were initially selected and subsequently filtered using relevant Japanese keywords such as “cancer,” “tumor,” “malignant,” “leukemia,” and “myelodysplastic syndrome.” Irrelevant reports were excluded following a manual review by the authors.

For comparison, we also reviewed reports of suicides or suicide attempts by patients with physical illnesses other than cancer. We first extracted reports categorized under “patient suicide” or “suicide attempt.” Reports originating from psychiatry or emergency departments were then excluded, as many incidents in emergency departments involved patients who had attempted suicide outside the hospital before admission. Finally, cancer‐related reports were excluded from the remaining data.

### 2.3 Topic Modeling

To provide an overview of the reports, we conducted topic modeling using BERTopic, a model designed for generating topics from text data, based on the Bidirectional Encoder Representations from Transformers (BERT), a transformer‐based large language model [21]. BERTopic has been widely applied in recent medical studies, including those on suicide [22].

We analyzed specific columns from the CSV files containing accident details (translated from Japanese as “Purpose of medical treatment,” “Incident details,” and “Overview of contributing factors”). Free‐text entries from these columns were split into sentences using punctuation as delimiters, and non‐informative sentences (e.g., “none”) were removed. BERTopic was then applied to the remaining sentences.

Similarly, we analyzed another column (translated from Japanese as “Improvement measures”) that contained countermeasures suggested by the medical staff. Text from this column was also split into sentences and processed using BERTopic.

To increase interpretability, we adjusted the number of topics using K‐means clustering. Two authors (K.K. and Y.U.) reviewed the sentences in each cluster, refined the number of clusters, and assigned topic names based on representative sentences and keywords.

These analyses were conducted using Python 3.11.5 and BERTopic 0.15.0, employing a pre‐trained Japanese model (paraphrase‐multilingual‐MiniLM‐L12‐v2).

### 2.4 OpenAI API Labeling

Research has demonstrated that large language models, such as ChatGPT, can accurately extract medical information from free‐text data, often achieving human‐level accuracy while reducing errors [13, 14, 15, 16, 17, 18, 19, 20]. For this study, the GPT‐4o model from the OpenAI API was used to label the content of the reports [23]. For instance, the following prompt was used to determine whether a patient exhibited signs of depressive symptoms before attempting or committing suicide:

The following sentences describe an inpatient who committed or attempted suicide. Based on these sentences, please indicate whether this patient presented with depressive symptoms BEFORE committing or attempting suicide. Please answer only with “Yes” (when they presented with depressive symptoms in all likelihood) or “No” (when they did not present with it, presented with it only after committing or attempting suicide, or it cannot be determined). The sentences are [input from each report].

Text from relevant columns (i.e., “Purpose of medical treatment,” “Incident details,” and “Overview of contributing factors”), as well as the patient's primary diagnosis, were included in the prompt to determine the label for patients' pre‐incident characteristics. In addition, text from the column “Improvement measures” was included in the prompt to determine the proposed action plans. We tested the prompts on several reports and adjusted them for accurate labeling. Table S1 provides the full list of prompts used. The labels were determined based on the generated topics (representative sentences and keywords), relevant literature [9, 10, 11], and a Japanese book summarizing inpatient suicide prevention strategies [24].

As a representative evaluation of the prompt labels, we randomly selected 20 cases, and a supervising psychiatrist (last author, Y.U.) assessed the presence or absence of depressive symptoms in these texts. We then calculated the crude agreement rate and the kappa coefficient for these assessments.

These labeling analyses were performed using Python 3.11.5 and OpenAI 1.2.4, with the “gpt‐4o” model from the OpenAI API. The role was set to “user,” and the temperature parameter was fixed at 0.0 to ensure the selection of the most probable response. Labeling was conducted between July 26 and September 10, 2024.

### 2.5 Association Between Patients' Characteristics and Action Plans

To examine the relationship between patients' pre‐incident characteristics and the proposed countermeasures, we conducted multivariate logistic regression analyses. Several characteristics before the incidents were selected as explanatory variables based on discussions among the authors. The outcome variables were the labels of the proposed countermeasures. Logistic regression models were developed for each outcome.

## 3 Results

### 3.1 Overview of the Reports

A total of 213 reports involving suicides (*N* = 134) and suicide attempts (*N* = 79) among patients with cancer were analyzed, encompassing 3306 sentences describing conditions both before and after the incidents. Table 1 outlines the identified topics, with the number of clusters fixed at 10. These topics include mental and physical distress, symptom deterioration, suicide ideation, nursing records, and post‐incident documentation.

**TABLE 1:** Overview of the reports.

No.	No. of sentences	No. of reports (%)	Topic name	Representative sentence (translated from Japanese into English)
0	476	175 (82.2%)	Mental and physical distress	The patient, undergoing chemotherapy for ovarian cancer with peritoneal dissemination and bone metastasis and awaiting surgery, had expressed her feelings less frequently since her first visit to our hospital. She often said things like, “I'm sorry” or “I hope I'm not inconveniencing others by being hospitalized”
1	391	161 (75.6%)	Symptom deterioration and medication	7:20—The patient's cancer‐related pain intensified, and medication was administered as prescribed
2	382	162 (76.1%)	Cancer treatment	The patient had undergone a right thoracotomy and laparotomy for a near‐total esophagectomy, aiming for a curative outcome, as there was a possibility of metastasis from stage I esophageal cancer (T1, N0, M0)
3	361	133 (62.4%)	Various nursing records	21:00—The nurse visited the patient's room to turn off the lights
4	358	173 (81.2%)	Suicidal ideation	The previous day, the patient had said, “I want to die,” prompting her second daughter to visit during the night
5	311	132 (62.0%)	Accident witnessing	10:05—The nurse visited the room and found the patient was cutting the abdomen with a fruit knife (blade length 9 cm)
6	308	139 (65.3%)	Appearance during hospitalization	14:40—The patient requested assistance in cleaning up after urinating, and the staff member attended to the request
7	277	120 (56.3%)	Resuscitation	Resuscitation efforts, including endotracheal intubation and adrenaline administration, were performed, but the patient's heartbeat did not resume
8	227	91 (42.7%)	Wound treatment	At 2:15 a.m., the physician arrived on the ward and performed suturing after sedation (four stitches). The cut was approximately 5 cm long, reaching the muscle layer partially, but most of the incision was superficial, about 1 mm deep, to the subcutaneous layer
9	215	103 (48.4%)	Post‐accident communication with family and police	The physician and police explained the incident to the family
Total	3306	213		

Table 2 presents topics generated from 568 sentences related to prevention countermeasures proposed by the involved medical staff, where the number of clusters was fixed at 4. The topics include information sharing with other professionals and families, measures regarding dangerous items and facilities, strategies for addressing mental distress, and efforts to reinforce suicide prevention awareness.

**TABLE 2:** Overview of the reports on prevention measures proposed by medical staff.

No.	No. of sentences	No. of reports (%)	Topic name	Representative sentence (translated from Japanese into English)
0	168	106 (49.8%)	Information sharing with other professionals and family	Doctors, nurses, the palliative care team, and the patient's family will work closely together to share information and prevent accidents
1	155	102 (47.9%)	Measures regarding dangerous items and facilities	Although patients with delirium were previously restricted from possessing dangerous items after family consultation, bringing in such items will now be strictly prohibited. At admission orientation, staff will check whether items like scissors have been brought in
2	131	100 (46.9%)	Measures for mental distress	To support the mental health of patients with cancer, ward nurses will actively consult with certified nurses, specialized nurses, and the palliative care team
3	114	86 (40.4%)	Reinforcement of suicide prevention awareness	To raise staff awareness about suicide prevention, the hospital will re‐announce guidelines, including recommendations for prevention, post‐event response, policies, and the suicide prevention manual
Total	568	213		

### 3.2 Demographics and Labels of the Reports

Table 3 summarizes the demographic and medical information. Table 4 presents all the conditions before the incident and the preventive countermeasures proposed by the medical staff, which were labeled using the OpenAI API with the prompt listed in Table S1. A crude agreement rate of 0.85 and a kappa coefficient of 0.69 were observed between the large language model‐generated labels and the supervising psychiatrist's assessments for depressive symptoms in 20 randomly selected cases, indicating “good” agreement [25]. For comparison, there were 214 reports containing patients with physical diseases other than cancer.

**TABLE 3:** Descriptive statistics of reports.

	Cancer (*N* = 213)	Non‐cancer (*N* = 214)
Age, *N* (%)		
10–19	0 (0.0)	5 (2.3)
20–29	1 (0.5)	12 (5.6)
30–39	5 (2.3)	18 (8.4)
40–49	17 (8.0)	18 (8.4)
50–59	31 (14.6)	22 (10.3)
60–69	53 (24.9)	33 (15.4)
70–79	79 (37.1)	55 (25.7)
80–89	26 (12.2)	46 (21.5)
90–99	1 (0.5)	5 (2.3)
Sex, *N* (%)		
Male	159 (74.6)	130 (60.7)
Female	54 (25.4)	84 (39.3)
Days of occurrence, *N* (%)		
Monday	33 (15.5)	21 (9.8)
Tuesday	24 (11.3)	27 (12.6)
Wednesday	29 (13.6)	25 (11.7)
Thursday	27 (12.7)	28 (13.1)
Friday	31 (14.6)	35 (16.4)
Saturday	29 (13.6)	42 (19.6)
Sunday	40 (18.8)	36 (16.8)
Time of occurrence, *N* (%)		
0:00–1:59	19 (8.9)	8 (3.7)
2:00–3:59	18 (8.5)	17 (7.9)
4:00–5:59	8 (3.8)	17 (7.9)
6:00–7:59	30 (14.1)	28 (13.1)
8:00–9:59	9 (4.2)	18 (8.4)
10:00–11:59	18 (8.5)	14 (6.5)
12:00–13:59	14 (6.6)	17 (7.9)
14:00–15:59	17 (8.0)	23 (10.7)
16:00–17:59	25 (11.7)	20 (9.3)
18:00–19:59	14 (6.6)	15 (7.0)
20:00–21:59	20 (9.4)	19 (8.9)
22:00–23:59	15 (7.0)	12 (5.6)
Unknown	6 (2.8)	6 (2.8)
Outcome, *N* (%)		
Death	134 (62.9)	105 (49.1)
Attempt or unknown	79 (37.1)	109 (50.9)
Accident location, *N* (%)		
Patient room	132 (62.0)	137 (64.0)
Toilet	14 (6.6)	19 (8.9)
Bathroom	5 (2.3)	0 (0.0)
Stairs	3 (1.4)	5 (2.3)
Outside the hospital	33 (15.5)	27 (12.6)
Other/Unknown	26 (12.2)	26 (12.2)
Involved personnel, *N* (%)		
Doctor	55 (25.8)	34 (15.9)
Nurse	147 (69.0)	172 (80.4)
Others	11 (5.2)	8 (3.7)
Years of experience of involved personnel, median (range)	8 (0–38)	9 (0–36)
Method of suicide, *N* (%)		
Hanging	103 (48.4)	114 (53.3)
Jumping	52 (24.4)	41 (19.2)
Stabbing/Cutting	37 (17.4)	24 (11.2)
Overdose	3 (1.4)	21 (9.8)
Other/Unknown	18 (8.5)	14 (6.5)
Tools for hanging, *N*		
Medical tubes	9	4
Medical equipment power/control cords	21	28
Personal power cords	16	21
Clothing	15	20
Non‐clothing household items	22	12
Others or not specified	20	29
Cancer type, *N* (%)		
Nervous system	8 (3.8)	N.A.
Head and neck	31 (14.6)	
Breast	5 (2.3)	
Respiratory	43 (20.2)	
Esophagus	16 (7.5)	
Gastrointestinal	35 (16.4)	
Liver	5 (2.3)	
Biliary/Pancreatic	16 (7.5)	
Gynecological	17 (8.0)	
Urological	12 (5.6)	
Hematological	22 (10.3)	
Others	3 (1.4)	
Cancer stage, *N* (%)		
Diagnosis	4 (1.9)	N.A.
Treatment (curative)	72 (33.8)	
Treatment (palliative)	125 (58.7)	
Treatment (unknown)	11 (5.2)	
Follow‐up	1 (0.5)	
Type of physical disease, *N* (%)		
Infectious disease	N.A.	6 (2.8)
Endocrine and metabolic		11 (5.1)
Nervous system		56 (26.2)
Respiratory system		32 (15.0)
Cardiovascular system		14 (6.5)
Gastrointestinal system		29 (13.6)
Genitourinary system		17 (7.9)
Skin and subcutaneous tissue		1 (0.5)
Musculoskeletal and connective tissue		31 (14.5)
Other/Unknown		17 (7.9)

**TABLE 4:** Conditions before incident and proposed countermeasures for prevention.

	Cancer (*N* = 213)	Non‐cancer (*N* = 214)
Mental conditions before the incident, *N* (%)		
Mental and psychological care by specialists	51 (23.9)	62 (29.0)
Use of psychotropic medication	64 (30.0)	70 (32.7)
Depressive symptoms	106 (49.8)	92 (43.0)
Anxiety	123 (57.7)	105 (49.1)
Insomnia	40 (18.8)	29 (13.6)
Delirium	27 (12.7)	26 (12.1)
Schizophrenia	4 (1.9)	7 (3.3)
Expression of suicidal ideation	74 (34.7)	73 (34.1)
Expression of desire for euthanasia	16 (7.5)	15 (7.0)
Physical conditions before the incident, *N* (%)		
Palliative care by specialists	29 (13.6)	2 (0.9)
Uncontrolled pain	55 (25.8)	25 (11.7)
Brain metastasis of cancer	9 (4.2)	N.A.
Pneumonia	14 (6.6)	14 (6.5)
Diabetes	8 (3.8)	19 (8.9)
Oral or pharyngeal symptoms	52 (24.4)	10 (4.7)
Loss of appetite	34 (16.0)	25 (11.7)
Dyspnea	27 (12.7)	27 (12.6)
Gastrostomy	4 (1.9)	1 (0.5)
Stoma	7 (3.3)	2 (0.9)
Opioid administration	29 (13.6)	1 (0.5)
Steroid administration	7 (3.3)	15 (7.0)
Antibiotic administration	18 (8.5)	18 (8.4)
Social and other conditions before the incident, *N* (%)		
Insufficient family support	53 (24.9)	69 (32.2)
Financial distress	3 (1.4)	11 (5.1)
Proposed countermeasures, *N* (%)		
Assessment and treatment of mental distress	114 (53.5)	132 (61.7)
Assessment and treatment of physical distress	28 (13.1)	11 (5.1)
Improvement in communication among medical staff	105 (49.3)	93 (43.5)
Information sharing with family	55 (25.8)	54 (25.2)
Preparation and use of internal manuals	27 (12.7)	33 (15.4)
Measures to prevent bringing in dangerous items	35 (16.4)	37 (17.3)
Measures against unauthorized leaving from the hospital	9 (4.2)	13 (6.1)
Measures for the building (e.g., windows, rooms)	41 (19.2)	41 (19.2)

Notably, about 50% of patients in both the cancer and non‐cancer groups exhibited depressive symptoms, and more than 30% had expressed suicidal ideation before the incident. In addition, approximately half of the patients exhibited anxiety, and around 7% expressed a desire for euthanasia in both groups. However, less than 30% of patients in both groups received specialized mental or psychological care. Additionally, more than 10% of patients in each group appeared to experience delirium before the incident. Among the patients with cancer, approximately one‐fourth experienced uncontrolled pain. Only 13.6% of cases involved specialized palliative care teams, and among these, about half (*N* = 15) continued to experience uncontrolled pain.

The most frequently proposed preventive measure in both groups was the assessment and treatment of mental distress. This was followed by recommendations for improving communication among medical staff and information sharing with the patient's families.

Overall, there were no notable differences between the cancer and non‐cancer groups, although the non‐cancer group tended to include slightly younger patients. Specialized palliative care interventions were applied in 13.6% of cancer cases but in only 0.9% (2 cases) of the non‐cancer group. A multivariate logistic regression analysis classifying patients as cancer or non‐cancer, using key pre‐incident labels as variables, showed that palliative care intervention and uncontrolled pain were significantly associated with cancer (see Table S2 for details), while no other significant differences were found between the two groups.

Table S3 compares the findings of this study with data from the National Cancer Registry [2], which includes all Japanese patients with cancer who died by suicide, both inside and outside hospitals, within 2 years of diagnosis. Notably, this study had a 4.2‐fold higher proportion of patients with head and neck cancer. Table S4 presents pre‐incident conditions and proposed countermeasures for patients with head and neck cancer, showing no substantial differences from the overall results.

### 3.3 Association Between Patients' Characteristics and Action Plans

**FIGURE 1:**
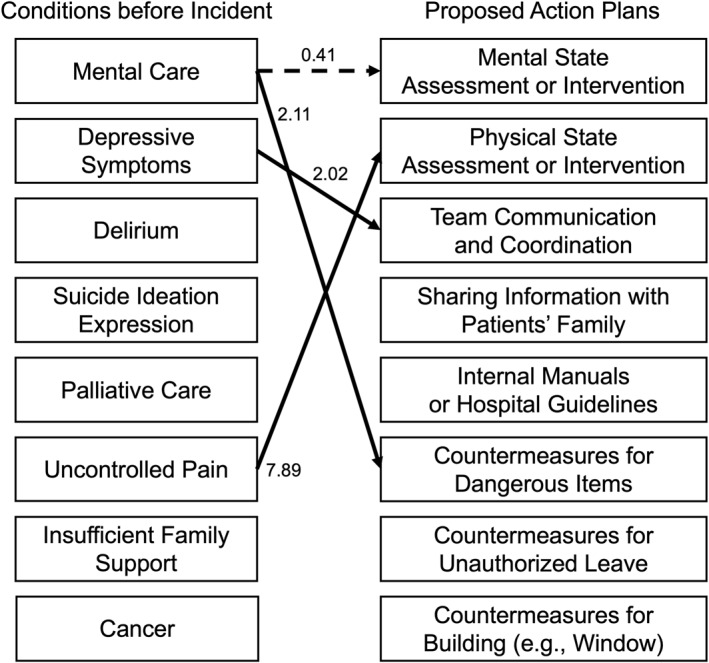
Association between pre‐incident conditions and proposed action plans. Only odds ratios with a *p*‐value < 0.01 are shown. Solid arrows represent positive associations, while the dotted arrow indicates a negative association.

Figure 1 presents odds ratios (ORs) with *p*‐values of < 0.01 for each multivariate logistic regression model. Table S5 provides the ORs and their confidence intervals (CIs) for each model. Key findings include: (1) Assessing and treating mental distress was less likely to be proposed for patients already receiving mental care (OR, 0.41; 95% CI, 0.25–0.65; *p* < 0.001). Conversely, reports for patients without prior care were more likely to include such recommendations. (2) For patients who had received mental care before the incident, staff were more likely to propose measures addressing dangerous items (OR, 2.11; 95% CI, 1.19–3.70; *p* = 0.009). (3) In cases where patients experienced poorly controlled pain, staff were more likely to suggest evaluating and treating physical distress (OR, 7.89; 95% CI, 3.71–17.24; *p* < 0.001). (4) Incidents involving patients who presented with depressive symptoms were more likely to include suggestions for improving communication among medical staff (OR, 2.02; 95% CI, 1.28–3.20; *p* = 0.003). Notably, the variable indicating cancer or other physical diseases was not significantly associated with any proposed countermeasures.

## 4 Discussion

This study comprehensively analyzed nationwide Japanese medical incident reports of suicides and suicide attempts among hospitalized patients with cancer, as well as those with other physical diseases, in major hospitals throughout Japan using large language models. This approach enabled a broader investigation of patients' mental and physical conditions, as well as incident‐based countermeasures, which have only been partially examined in previous research. While exploratory, the analysis provided novel insights into the association between patients' pre‐incident conditions and the proposed countermeasures, an area previously unexamined.

The data analyzed in the present study involved a 4.2‐fold higher proportion of head and neck cancer compared to that in a previous study using the National Cancer Registry [2], which included all suicides among patients with cancer in Japan, with 70% of these occurring at home. Patients with head and neck cancer may face an elevated risk of in‐hospital suicide, possibly related to distress from the side effects of treatment, including esthetic and functional impairments, difficulty swallowing, and ototoxicity [26]. This study found no substantial differences in conditions such as depressive symptoms or the expression of suicidal ideation between patients with head and neck cancer and those with other types of cancers. The discrepancy in cancer‐type proportions may be related to differences in the observation periods between the current study and the study using the National Cancer Registry, which focused on suicides within 2 years of diagnosis [2]. Aside from the proportion of cancer type, no notable differences were observed between the present study and the National Cancer Registry‐based research. However, the National Cancer Registry lacks sufficient data on factors potentially related to suicide, including medical treatment, hospital environment, and psychological support, making comparisons in these aspects difficult. Further research is needed to assess the generalizability of the present findings and the differences between suicide in inpatients and the general cancer population.

Approximately 50% of patients with cancer in this study exhibited depressive symptoms pre‐incident. Although determining a diagnosis of depression was challenging in the present study, this proportion of depressive symptoms is much higher than the general prevalence of depression among patients with cancer [27]. This percentage may even be an underestimation due to the frequent underdiagnosis of depression in patients with cancer [28]. Additionally, over 30% of patients expressed suicidal ideation. Furthermore, findings suggesting psychological issues, such as anxiety and expression of desire for euthanasia, had been identified before the incident. However, only 24% received specialist mental care. This limited access to psychiatric consultation in medical settings is consistent with previous findings from Japan [10]. Logistic regression revealed that recommendations for assessing and treating mental distress were more frequently proposed for patients who had not previously received mental care. This suggests that the stigma surrounding psychiatric consultation may affect both patients and healthcare providers [29]. Further research should explore barriers to accessing psychiatry consultations in medical settings.

Notably, 20%–30% of patients who had received psychiatric care still experienced suicide‐related incidents. Future studies should investigate the quality and intensity of psychiatric interventions in these cases. Logistic regression analysis revealed that countermeasures addressing dangerous items were more likely to be proposed for patients under psychiatric care, suggesting that combining mental health support with environmental safety measures could be beneficial.

Delirium was another important finding, with over 10% of patients in both cancer and non‐cancer groups likely experiencing this condition, indicative of confusion. The high prevalence of delirium may reflect Japan's status as one of the most aging societies globally [30]. Delirium has been observed as a potential contributor to fatal incidents [31], and it often coexists with suicidal ideation in patients with cancer [32]. Appropriate prevention and management of delirium, based on psycho‐oncology guidelines [33], could serve as an important suicide prevention measure. Delirium may have been underestimated in this study due to factors such as hypoactive delirium being overlooked [34] and misdiagnosed as depression [35]; thus, the actual prevalence may have been even higher than that detected in this study, given the large proportion of palliative cases. This study highlights a new perspective on the potential involvement of delirium in hospital suicides, which has not been previously discussed in related studies [9, 10, 11].

Among patients with cancer, 25.8% experienced uncontrolled pain, and only 13.6% received specialized palliative care. Inadequate intervention for physical distress likely contributed to the incidents. Logistic regression analysis showed that reports involving uncontrolled pain were more likely to include physical interventions as countermeasures, possibly reflecting healthcare providers' regret. Previous research has underscored the importance of addressing physical distress in patients with cancer [11]. Additionally, physical assessments are also essential for managing delirium [36]. These findings highlight the potential effectiveness of physical care in suicide prevention.

Communication among medical staff was identified as an area for improvement, particularly in cases where patients presented with depressive symptoms before the incident. This implies that depressive symptoms, an evident risk factor, may not have been adequately communicated by the care team. Information recorded in patient charts may have been overlooked by other staff members. Enhancing communication through multidisciplinary conferences and active information sharing among staff may be essential. Assertive case management has been shown to reduce suicide attempts [37]. Additionally, implementing reminders linked to patients' medical charts could help staff monitor high‐risk individuals more effectively.

The number of reports involving patients with cancer and non‐cancer physical disease was nearly identical, consistent with a previous study in Japan [10]. The non‐cancer group included younger patients and had less involvement from palliative care teams. In Japan, palliative care is primarily focused on patients with cancer [38], but extending these services to patients with other physical diseases may be beneficial. The logistic regression revealed no significant differences in countermeasures suggestions between the cancer and non‐cancer groups, suggesting that similar action plans were considered effective. While suicide prevention guidelines for inpatients with cancer have been developed [5], extending similar guidelines to other physical diseases could also be valuable.

### 4.1 Implications

To summarize these findings and considerations, several measures appear potentially useful for suicide prevention among hospitalized patients with cancer. These include thorough mental health assessments and appropriate consultation, particularly for patients with depressive symptoms, addressing the presence of dangerous items, proper management of delirium, sufficient pain control, and ensuring effective communication among medical staff when patients present with depressive symptoms. While these measures are still at the proposal stage from the involved staff, they may be valuable for clinicians.

### 4.2 Study Limitations

This study has several limitations. First, the quantity and quality of information in each report varied, making it challenging to assess undocumented or vaguely documented details. Second, the extent of compliance in reporting attempted cases remains unclear, leading to potential reporting bias in these data. Notably, fewer suicide attempts (*N* = 79) were reported than suicides (*N* = 134) in this study, which may contradict reports from Japan suggesting that suicide attempts are more common than completed suicides [39]. Third, while labels such as mental health intervention were made, the specific details, such as the frequency or form of care, remain unexamined. Fourth, whereas we confirmed good agreement with experts for depressive symptoms regarding the labels generated using the OpenAI API, we were unable to verify all labels across the entire dataset. Fifth, no data were available from patients without suicide‐related incidents, preventing an investigation into the impact of preceding factors on these events. Future studies should aim to include such control groups for comparison. Sixth, the countermeasures discussed are based on suggestions made by reporters, and their effectiveness remains largely unexplored. Seventh, appropriate preventive measures often depend on hospital resources, yet these background factors could not be evaluated in this study. Eighth, while relationships between pre‐incident conditions and proposed countermeasures were analyzed, caution is required due to the multiple statistical tests conducted. Lastly, further refinement is needed to extract conceptual information, such as whether patients had received distressing news, commonly referred to as “bad news,” before the incident.

### 4.3 Conclusion

This study examined suicides among inpatients with cancer by analyzing patient safety reports from major hospitals across Japan using large language models. The analysis offers insights into strategies for preventing suicides in hospitalized patients with cancer. These strategies include providing mental care for patients with depressive symptoms or suicidal ideation, addressing physical distress in those experiencing uncontrolled pain, managing delirium appropriately, and enhancing communication among medical staff when patients present with depressive symptoms. Many of these strategies are consistent with those proposed in previous reports. Additionally, this study identified the need for more attention to delirium, which has been unexplored in existing research. These strategies could also be valuable for patients with physical diseases other than cancer.

## Author Contributions

**Ken Kurisu:** conceptualization, formal analysis, data curation, methodology, software, visualization, writing – original draft preparation, writing – review and editing. **Maiko Fujimori:** conceptualization, funding acquisition, project administration, supervision, writing – review and editing. **Saki Harashima:** writing – review and editing. **Masako Okamura:** project administration, writing – review and editing. **Kazuhiro Yoshiuchi:** writing – review and editing. **Yosuke Uchitomi:** supervision, writing – review and editing.

## Conflicts of Interest

The authors declare no conflicts of interest.

## Supporting information

Supporting Information S1

## Data Availability

The dataset used in this study is available on the Japan Council for Quality Health Care website.
