# Supplementary material for: Exploratory Analysis of Nationwide Japanese Patient Safety Reports on Suicide and Suicide Attempts Among Inpatients With Cancer Using Large Language Models
Source: Psychooncology. 2025 May 4;34(5):e70150. doi: 10.1002/pon.70150 (PMC12050354; doi:10.1002/pon.70150)
Supplement: Supplementary file 1 — Supporting Information S1 [file PON-34-e70150-s001.docx]

**Supplementary Table 1. Lists of prompt**

| Label | Prompt |
| --- | --- |
| Demographics |  |
| Method of Suicide | The following sentences describe an inpatient who committed or attempted suicide. Please select the patient’s method of suicide from the following four options: Hanging, Jumping, Stabbing/Cutting, Overdose, Other/Unknown. Answer only with options. The sentences are …. |
| Tools of Hanging | The following sentences describe an inpatient who committed or attempted suicide by hanging. Please answer the patient’s tool of hanging, such as intravenous line. Answer only with the tool briefly. The sentences are …. |
| Accident Location  (Outside the Hospital) | The following sentences describe an inpatient who committed or attempted suicide by hanging. Please answer whether the patient committed or attempted suicide outside the hospital. Please answer only with 'Yes' (outside the hospital) or 'No' (inside the hospital). The sentences are …. |
| Cancer Type | The following sentences describe an inpatient with cancer who committed or attempted suicide. Please select the patient’s primary tumor site from the following options: 'Blood and lymph', 'Brain, nerves, and eyes', 'Breast', 'Chest', 'Esophagus', 'Gastrointestinal tract', 'Gynecology', 'Head and neck', 'Liver', 'Pancreas and gallbladder', 'Urinary organs', 'Other'. Answer only with options. The sentences are …. |
| Cancer Stage | The following sentences describe an inpatient with cancer who committed or attempted suicide. Please select the patient’s stage in cancer care from the following options: 'Diagnosis', 'Treatment (curative)', 'Treatment (palliative)', 'Treatment (unknown)', '5‐year follow‐up', 'Unable to determine' Answer only with options. The sentences are …. |
| Type of Physical Diseases | The following sentences describe an inpatient with cancer who committed or attempted suicide. Please select the patient’s main physical disease type from the following options: 'Infection', 'Endocrine and Metabolic', 'Nervous System', 'Respiratory', 'Cardiovascular', 'Gastrointestinal', 'Genitourinary', 'Skin and Subcutaneous Tissue', 'Musculoskeletal and Connective Tissue', 'Other or Unknown'. Please answer only with options. The sentences are …. |
| Mental Conditions before incident |  |
| Mental and psychological care by specialists | The following sentences describe an inpatient who committed or attempted suicide. Based on these sentences, please indicate whether this patient received specialized treatment or examination for mental health issues from a specialist BEFORE committing or attempting suicide. Please answer only with 'Yes' (when they received such treatment or examination in all likelihood) or 'No' (when they did not receive it, they received it only after committing or attempting suicide, or it cannot be determined). The sentences are …. |
| Use of psychotropic medication | The following sentences describe an inpatient who committed or attempted suicide. Based on these sentences, please indicate whether this patient took psychotropic drugs BEFORE committing or attempting suicide. Please answer only with 'Yes' (when they did in all likelihood) or 'No' (when they did not, they took psychotropics only after committing or attempting suicide, or it cannot be determined). The sentences are …. |
| Depressive symptoms | The following sentences describe an inpatient who committed or attempted suicide. Based on these sentences, please indicate whether this patient presented with depressive symptoms BEFORE committing or attempting suicide. Please answer only with 'Yes' (when they presented with depressive symptoms in all likelihood) or 'No' (when they did not present with it, they presented with it only after committing or attempting suicide, or it cannot be determined). The sentences are …. |
| Anxiety | The following sentences describe an inpatient who committed or attempted suicide. Based on these sentences, please indicate whether this patient presented with anxiety BEFORE committing or attempting suicide. Please answer only with 'Yes' (when they presented with anxiety in all likelihood) or 'No' (when they did not present with it, they presented with it only after committing or attempting suicide, or it cannot be determined). The sentences are …. |
| Insomnia | The following sentences describe an inpatient who committed or attempted suicide. Based on these sentences, please indicate whether this patient presented with insomnia BEFORE committing or attempting suicide. Please answer only with 'Yes' (when they presented with insomnia in all likelihood) or 'No' (when they did not present with it, they presented with it only after committing or attempting suicide, or it cannot be determined). The sentences are …. |
| Delirium | The following sentences describe an inpatient who committed or attempted suicide. Based on these sentences, please indicate whether this patient presented with delirium BEFORE committing or attempting suicide. Please answer only with 'Yes' (when they presented with delirium in all likelihood) or 'No' (when they did not present with it, they presented with it only after committing or attempting suicide, or it cannot be determined). The sentences are …. |
| Schizophrenia | The following sentences describe an inpatient who committed or attempted suicide. Based on these sentences, please indicate whether this patient presented with schizophrenia. Please answer only with 'Yes' (when they presented with schizophrenia in all likelihood) or 'No' (when they did not present with it, or it cannot be determined). The sentences are …. |
| Expression of suicidal ideation | The following sentences describe an inpatient who committed or attempted suicide. Based on these sentences, please indicate whether this patient expressed suicidal thoughts BEFORE committing or attempting suicide. Please answer only with 'Yes' (when they expressed it in all likelihood) or 'No' (when they did not, they did it only after committing or attempting suicide, or it cannot be determined). The sentences are …. |
| Expression of desire for euthanasia | The following sentences describe an inpatient who committed or attempted suicide. Based on these sentences, please indicate whether this patient expressed a desire for euthanasia BEFORE committing or attempting suicide. Please answer only with 'Yes' (when they expressed it in all likelihood) or 'No' (when they did not, they did it only after committing or attempting suicide, or it cannot be determined). The sentences are …. |
| Physical condition before incident |  |
| Palliative care by specialists | The following sentences describe an inpatient who committed or attempted suicide. Based on these sentences, please indicate whether this patient received specialized palliative care from a specialist BEFORE committing or attempting suicide. Please answer only with 'Yes' (when they received such care in all likelihood) or 'No' (when they did not receive it, they received it only after committing or attempting suicide, or it cannot be determined). The sentences are …. |
| Uncontrolled pain | The following sentences describe an inpatient who committed or attempted suicide. Based on these sentences, please indicate whether this patient expressed uncontrolled pain BEFORE committing or attempting suicide. Please answer only with 'Yes' (when they expressed such pain in all likelihood) or 'No' (when they did not express it, they expressed it only after committing or attempting suicide, or it cannot be determined). The sentences are …. |
| Brain metastasis | The following sentences describe an inpatient who committed or attempted suicide. Based on these sentences, please indicate whether this patient presented with brain metastasis of cancer. Please answer only with 'Yes' (when they presented with it in all likelihood) or 'No' (when they did not present with it, or it cannot be determined). The sentences are …. |
| Pneumonia | The following sentences describe an inpatient who committed or attempted suicide. Based on these sentences, please indicate whether this patient presented with pneumonia. Please answer only with 'Yes' (when they presented with it in all likelihood) or 'No' (when they did not present with it, or it cannot be determined). The sentences are …. |
| Diabetes | The following sentences describe an inpatient who committed or attempted suicide. Based on these sentences, please indicate whether this patient presented with diabetes. Please answer only with 'Yes' (when they presented with diabetes.py in all likelihood) or 'No' (when they did not present with it, or it cannot be determined). The sentences are …. |
| Oral or pharyngeal symptoms | The following sentences describe an inpatient who committed or attempted suicide. Based on these sentences, please indicate whether this patient presented with oral or pharyngeal symptoms BEFORE committing or attempting suicide. Please answer only with 'Yes' (when they presented with oral and pharyngeal symptoms in all likelihood) or 'No' (when they did not present with it, they presented with it only after committing or attempting suicide, or it cannot be determined). The sentences are …. |
| Loss of appetite | The following sentences describe an inpatient who committed or attempted suicide. Based on these sentences, please indicate whether this patient presented with decreased appetite or food intake. Please answer only with 'Yes' (when they presented with it in all likelihood) or 'No' (when they did not present with it, or it cannot be determined). The sentences are …. |
| Dyspnea | The following sentences describe an inpatient who committed or attempted suicide. Based on these sentences, please indicate whether this patient presented with dyspnea BEFORE committing or attempting suicide. Please answer only with 'Yes' (when they presented with dyspnea in all likelihood) or 'No' (when they did not present with it, they presented with it only after committing or attempting suicide, or it cannot be determined). The sentences are …. |
| Gastrostomy | The following sentences describe an inpatient who committed or attempted suicide. Based on these sentences, please indicate whether this patient had gastrosomy. Please answer only with 'Yes' (when they did in all likelihood) or 'No' (when they did not, or it cannot be determined). The sentences are …. |
| Stoma | The following sentences describe an inpatient who committed or attempted suicide. Based on these sentences, please indicate whether this patient had colostomy (stoma). Please answer only with 'Yes' (when they did in all likelihood) or 'No' (when they did not, or it cannot be determined). The sentences are …. |
| Opioid administration | The following sentences describe an inpatient who committed or attempted suicide. Based on these sentences, please indicate whether this patient took opioid BEFORE committing or attempting suicide. Please answer only with 'Yes' (when they did in all likelihood) or 'No' (when they did not, they took opioid only after committing or attempting suicide, or it cannot be determined). The sentences are …. |
| Steroid administration | The following sentences describe an inpatient who committed or attempted suicide. Based on these sentences, please indicate whether this patient took steroid BEFORE committing or attempting suicide. Please answer only with 'Yes' (when they did in all likelihood) or 'No' (when they did not, they took steroid only after committing or attempting suicide, or it cannot be determined). The sentences are …. |
| Antibiotic administration | The following sentences describe an inpatient who committed or attempted suicide. Based on these sentences, please indicate whether this patient took antibiotics BEFORE committing or attempting suicide. Please answer only with 'Yes' (when they did in all likelihood) or 'No' (when they did not, or it cannot be determined). The sentences are …. |
| Social and other conditions before incident |  |
| Insufficient family support | The following sentences describe an inpatient who committed or attempted suicide. Based on these sentences, please indicate whether this patient had insufficient family support. Please answer only with 'Yes' (when they did in all likelihood) or 'No' (when they did not, or it cannot be determined). The sentences are …. |
| Financial distress | The following sentences describe an inpatient who committed or attempted suicide. Based on these sentences, please indicate whether this patient had financial distress. Please answer only with 'Yes' (when they did in all likelihood) or 'No' (when they did not, or it cannot be determined). The sentences are …. |
| Proposed counrtermeasures |  |
| Assessment and treatment of mental distress | The following sentences describe improvement measures for an inpatient who committed or attempted suicide. Do these sentences include the topic of mental state assessment or intervention? Please answer only with ‘Yes’ (if it includes it in all likelihood) or ‘No’ (if it does not or it cannot be determined). The sentences are …. |
| Assessment and treatment of physical distress | The following sentences describe improvement measures for an inpatient who committed or attempted suicide. Do these sentences include the topic of physical state assessment or intervention, such as pain management? Please answer only with ‘Yes’ (if it includes it in all likelihood) or ‘No’ (if it does not or it cannot be determined). The sentences are …. |
| Improvement in communication among medical staff | The following sentences describe improvement measures for an inpatient who committed or attempted suicide. Do these sentences include the topic of team communication or coordination? The sentences are …. |
| Information sharing with family | The following sentences describe improvement measures for an inpatient who committed or attempted suicide. Do these sentences include the topic of sharing information with patients' family? Please answer only with 'Yes' (if it includes it in all likelihood) or 'No' (if it does not or it cannot be determined). The sentences are …. |
| Preparation and use of internal manuals | The following sentences describe improvement measures for an inpatient who committed or attempted suicide. Do these sentences include the topic of internal manuals or hospital guidelines? Please answer only with ‘Yes’ (if it includes it in all likelihood) or ‘No’ (if it does not or it cannot be determined). The sentences are …. |
| Measures to prevent bringing in dangerous items | The following sentences describe improvement measures for an inpatient who committed or attempted suicide. Do these sentences include the topic of bringing in dangerous items? Please answer only with ‘Yes’ (if it includes it in all likelihood) or ‘No’ (if it does not or it cannot be determined). The sentences are …. |
| Measures against unauthorized leaving from the hospital | The following sentences describe improvement measures for an inpatient who committed or attempted suicide. Do these sentences include the topic of unauthorized discharge or leave from hospitals? Please answer only with ‘Yes’ (if it includes it in all likelihood) or ‘No’ (if it does not or it cannot be determined). The sentences are …. |
| Measures for the building (e.g., windows, rooms) | The following sentences describe improvement measures for an inpatient who committed or attempted suicide. Do these sentences include the topic of buildings, such as windows, locking, or rooms? Please answer only with 'Yes' (if it includes it in all likelihood) or 'No' (if it does not or it cannot be determined). The sentences are …. |

**Supplementary Table 2. Multivariate logistic regression model predicting cancer vs. non-cancer status based on pre-incident factors**

| Variables | Odds Ratio (95% CI) | P-value |
| --- | --- | --- |
| Mental Care | 0.63 (0.39-1.01) | 0.059 |
| Depressive symptoms | 1.40 (0.88-2.24) | 0.16 |
| Delirium | 1.03 (0.55-1.91) | 0.93 |
| Suicide Ideation Expression | 0.80 (0.48-1.32) | 0.38 |
| Palliative Care | 16.91 (4.78-107.97) | **< 0.001** |
| Uncontrolled Pain | 2.02 (1.17-3.53) | **0.012** |
| Insufficient Family Support | 0.69 (0.44-1.09) | 0.11 |

**Supplementary Table 3. Comparison of descriptive statistics with the patients with cancer who died by suicide within two years of diagnosis revealed by the National Cancer Registry**

|  | This study (N=213) | National Cancer Registry (N=660) |
| --- | --- | --- |
| Age, N (%)  20-39  40-49  50-59  60-69  70-79  80- | 6 (2.8)  17 (8.0)  31 (14.6)  53 (24.9)  79 (37.1)  27 (12.7) | 15 (2.3)  45 (6.8)  69 (10.5)  178 (27.0)  230 (34.8)  123 (18.6) |
| Sex, N (%)  Male  Female | 159 (74.6)  54 (25.4) | 473 (71.7)  187 (28.3) |
| Cancer Type, N (%)  Head and Neck  Breast  Respiratory  Esophagus  Gastrointestinal  Liver  Biliary/Pancreatic  Gynecological  Urological  Others | 31 (14.6)  5 (2.3)  43 (20.2)  16 (7.5)  35 (16.4)  5 (2.3)  16 (7.5)  17 (8.0)  12 (5.6)  33 (15.5) | 23 (3.5)  48 (7.3)  64 (9.7)  32 (4.8)  205 (31.1)  22 (3.3)  35 (5.3)  26 (3.9)  102 (15.5)  103 (15.6) |

**Supplementary Table 4. Descriptive statistics of patients with head and neck cancer**

|  | N=31 |
| --- | --- |
| Conditions before the incident, N (%)  Mental and psychological care by specialists  Use of psychotropic medication  Depressive symptoms  Insomnia  Delirium  Expression of suicidal ideation  Palliative care by specialists  Uncontrolled pain | 8 (25.8)  5 (16.1)  15 (48.4)  6 (19.4)  2 (6.5)  7 (22.6)  5 (16.1)  7 (22.6) |
| Proposed counrtermeasures, N (%)  Assessment and treatment of mental distress  Assessment and treatment of physical distress  Improvement in communication among medical staff  Information sharing with family  Preparation and use of internal manuals  Measures to prevent bringing in dangerous items  Measures against unauthorized leaving from the hospital  Measures for the building (e.g., windows, rooms) | 20 (64.5)  5 (16.1)  13 (41.9)  7 (22.6)  5 (16.1)  7 (22.6)  1 (3.2)  5 (16.1) |

**Supplementary Table 5. Multivariate logistic regression model on the relationship between each proposed counrtermeasures and conditions before incident**

| Variables | Odds Ratio (95% CI) | P-value |
| --- | --- | --- |
| Mental State Assessment or Intervention |  |  |
| Mental Care | 0.41 (0.25-0.65) | **< 0.01** |
| Depressive symptoms | 1.49 (0.94-2.38) | 0.093 |
| Delirium | 1.19 (0.65-2.22) | 0.58 |
| Suicide Ideation Expression | 1.24 (0.76-2.04) | 0.40 |
| Palliative Care | 1.37 (0.61-3.15) | 0.45 |
| Uncontrolled Pain | 1.04 (0.62-1.79) | 0.87 |
| Insufficient Family Support | 1.04 (0.67-1.64) | 0.85 |
| Cancer | 0.63 (0.41-0.95) | 0.028 |
| Physical State Assessment or Intervention |  |  |
| Mental Care | 0.29 (0.08-0.82) | 0.032 |
| Depressive symptoms | 1.93 (0.83-4.50) | 0.13 |
| Delirium | 0.99 (0.32-2.72) | 0.99 |
| Suicide Ideation Expression | 0.67 (0.27-1.61) | 0.38 |
| Palliative Care | 1.84 (0.57-5.61) | 0.29 |
| Uncontrolled Pain | 7.89 (3.71-17.24) | **< 0.01** |
| Insufficient Family Support | 1.81 (0.80-4.05) | 0.15 |
| Cancer | 1.83 (0.82-4.25) | 0.15 |
| Team Communication and Coordination |  |  |
| Mental Care | 0.65 (0.40-1.04) | 0.078 |
| Depressive symptoms | 2.02 (1.28-3.20) | **< 0.01** |
| Delirium | 1.10 (0.59-2.04) | 0.76 |
| Suicide Ideation Expression | 1.74 (1.07-2.83) | 0.025 |
| Palliative Care | 2.05 (0.88-5.02) | 0.10 |
| Uncontrolled Pain | 1.24 (0.73-2.12) | 0.42 |
| Insufficient Family Support | 1.42 (0.91-2.23) | 0.12 |
| Cancer | 1.09 (0.72-1.66) | 0.67 |
| Sharing Information with Patients’ Family |  |  |
| Mental Care | 0.76 (0.44-1.28) | 0.32 |
| Depressive symptoms | 1.10 (0.65-1.84) | 0.72 |
| Delirium | 1.03 (0.51-1.98) | 0.93 |
| Suicide Ideation Expression | 1.14 (0.66-1.96) | 0.63 |
| Palliative Care | 0.95 (0.37-2.27) | 0.91 |
| Uncontrolled Pain | 1.31 (0.73-2.29) | 0.35 |
| Insufficient Family Support | 1.38 (0.85-2.23) | 0.19 |
| Cancer | 1.00 (0.63-1.59) | 0.99 |
| Internal Manuals or Hospital Guidelines |  |  |
| Mental Care | 0.95 (0.49-1.79) | 0.89 |
| Depressive symptoms | 1.01 (0.52-1.93) | 0.98 |
| Delirium | 1.14 (0.47-2.49) | 0.76 |
| Suicide Ideation Expression | 1.33 (0.68-2.61) | 0.40 |
| Palliative Care | 2.94 (1.06-7.70) | 0.031 |
| Uncontrolled Pain | 0.63 (0.26-1.36) | 0.27 |
| Insufficient Family Support | 0.98 (0.52-1.79) | 0.94 |
| Cancer | 0.70 (0.38-1.26) | 0.24 |
| Countermeasures for Dangerous Items |  |  |
| Mental Care | 2.11 (1.19-3.70) | **< 0.01** |
| Depressive symptoms | 1.57 (0.86-2.85) | 0.14 |
| Delirium | 1.27 (0.58-2.59) | 0.53 |
| Suicide Ideation Expression | 0.80 (0.43-1.48) | 0.48 |
| Palliative Care | 1.12 (0.40-2.84) | 0.82 |
| Uncontrolled Pain | 1.37 (0.69-2.60) | 0.35 |
| Insufficient Family Support | 0.92 (0.50-1.64) | 0.79 |
| Cancer | 0.89 (0.51-1.53) | 0.66 |
| Countermeasures for Unauthorized Leave |  |  |
| Mental Care | 1.10 (0.40-2.85) | 0.85 |
| Depressive symptoms | 1.28 (0.45-3.82) | 0.65 |
| Delirium | 0.36 (0.02-1.83) | 0.33 |
| Suicide Ideation Expression | 2.47 (0.87-7.34) | 0.095 |
| Palliative Care | 2.30 (0.45-9.34) | 0.27 |
| Uncontrolled Pain | 0.61 (0.13-2.01) | 0.46 |
| Insufficient Family Support | 0.74 (0.25-1.91) | 0.55 |
| Cancer | 0.61 (0.22-1.55) | 0.31 |
| Countermeasures for Building (e.g., Window) |  |  |
| Mental Care | 1.22 (0.69-2.13) | 0.49 |
| Depressive symptoms | 0.75 (0.42-1.32) | 0.32 |
| Delirium | 1.60 (0.79-3.11) | 0.17 |
| Suicide Ideation Expression | 1.17 (0.64-2.15) | 0.61 |
| Palliative Care | 0.57 (0.16-1.65) | 0.34 |
| Uncontrolled Pain | 0.80 (0.39-1.55) | 0.53 |
| Insufficient Family Support | 0.96 (0.55-1.65) | 0.88 |
| Cancer | 1.13 (0.68-1.88) | 0.63 |
